# GABAergic Input From the Basal Forebrain Promotes the Survival of Adult-Born Neurons in the Mouse Olfactory Bulb

**DOI:** 10.3389/fncir.2020.00017

**Published:** 2020-04-23

**Authors:** Elizabeth Hanson, Jessica Swanson, Benjamin R. Arenkiel

**Affiliations:** ^1^Department of Molecular and Human Genetics, Baylor College of Medicine, Houston, TX, United States; ^2^Program in Developmental Biology, Baylor College of Medicine, Houston, TX, United States; ^3^Department of Neuroscience, Baylor College of Medicine, Houston, TX, United States; ^4^Jan and Dan Duncan Neurological Research Institute at Texas Children’s Hospital, Baylor College of Medicine, Houston, TX, United States

**Keywords:** adult neurogenesis, GABA, basal forebrain, olfaction, granule cells

## Abstract

A unique feature of the olfactory system is the continuous generation and integration of new neurons throughout adulthood. Adult-born neuron survival and integration is dependent on activity and sensory experience, which is largely mediated by early synaptic inputs that adult-born neurons receive upon entering the olfactory bulb (OB). As in early postnatal development, the first synaptic inputs onto adult-born neurons are GABAergic. However, the specific sources of early synaptic GABA and the influence of specific inputs on adult-born neuron development are poorly understood. Here, we use retrograde and anterograde viral tracing to reveal robust GABAergic projections from the basal forebrain horizontal limb of the diagonal band of Broca (HDB) to the granule cell layer (GCL) and glomerular layer (GL) of the mouse OB. Whole-cell electrophysiological recordings indicate that these projections target interneurons in the GCL and GL, including adult-born granule cells (abGCs). Recordings from birth-dated abGCs reveal a developmental time course in which HDB GABAergic input onto abGCs emerges as the neurons first enter the OB, and strengthens throughout the critical period of abGC development. Finally, we show that removing GABAergic signaling from HDB neurons results in decreased abGC survival. Together these data show that GABAergic projections from the HDB synapse onto immature abGCs in the OB to promote their survival through the critical period, thus representing a source of long-range input modulating plasticity in the adult OB.

## Introduction

Olfaction is a key sensory modality guiding behaviors from feeding to mating. To interpret olfactory information in different contexts, sensory processing adapts throughout life to an animal’s experience, and changes moment-to-moment based on context and behavioral state. Accordingly, the olfactory system is subject to potent top-down regulation (de Araujo et al., 2005). Additionally, the olfactory system features the ongoing generation and integration of adult-born neurons (Altman and Das, 1966; Hinds, 1968; Altman, 1969), endowing the olfactory bulb (OB) with unique forms of cellular and circuit plasticity.

In the adult brain, new neurons are continuously generated in the subventricular zone (SVZ) and then migrate along the rostral migratory stream (RMS) to the OB (Luskin, 1993; Lois and Alvarez-Buylla, 1994; Alvarez-Buylla and Garcıa-Verdugo, 2002). Once in the OB, they integrate primarily as GABAergic adult-born granule cells (abGCs) or periglomerular GABAergic interneurons (Luskin, 1993; Lois and Alvarez-Buylla, 1994). The continuous integration of immature neurons endows the OB with unique forms of structural and functional plasticity (Saghatelyan et al., 2005; Nissant et al., 2009; Lepousez et al., 2014). As adult-born neurons integrate, they undergo a critical period of enhanced synaptic plasticity (Kelsch et al., 2009). During this time they either integrate or undergo apoptosis (Najbauer and Leon, 1995). Ultimately, only about half of the adult-born neurons that enter the OB survive (Petreanu and Alvarez-Buylla, 2002; Winner et al., 2002; Yamaguchi and Mori, 2005). This process is influenced by sensory experience, with olfactory deprivation leading to decreased survival and enrichment leading to increased survival, integration, and maturation of receptive fields (Corotto et al., 1994; Fiske and Brunjes, 2001; Petreanu and Alvarez-Buylla, 2002; Rochefort et al., 2002; Alonso et al., 2006; Quast et al., 2016). Thus, adult neurogenesis establishes lasting changes in olfactory circuits that reflect sensory experience throughout life. Experience-dependent integration of abGCs, in turn, indicates that top-down inputs play a role in abGC development. Thus, it is critically important to determine the sources of early synaptic input onto adult-born neurons in the OB. To this end, rabies-based retrograde tracing from abGCs showed local OB inputs to young abGCs (Deshpande et al., 2013). A disadvantage of the rabies-based approach, however, is that it does not label all inputs equally for reasons that are yet undefined. It is possible in the context of early abGC development that rabies tracing can miss unconventional and/or immature inputs. Thus, it is necessary to take additional anatomical and functional approaches to investigate potential, early inputs onto abGCs that may influence abGC survival and maturation.

In the embryonic and postnatal developing brain, early tonic and synaptic GABA signals drive neuronal migration, maturation, and integration. Similarly, adult-born neurons express functional GABA receptors at all developmental time points (Belluzzi et al., 2003), and their proliferation, migration, and maturation, from the SVZ to the OB is guided by GABAergic cues (Stewart et al., 2002; Wang et al., 2003; Bolteus and Bordey, 2004; Liu et al., 2005; Pallotto et al., 2012). Importantly, the postnatal switch in GABA polarity from depolarizing to hyperpolarizing is replicated in OB adult-born neurons (Ben-Ari et al., 1989; Belluzzi et al., 2003; Carleton et al., 2003).

Despite the key role of GABA regulating multiple aspects of postnatal and adult-born neuron development, investigating the influence of GABAergic drive on abGC development has proven challenging. In particular, diverse GABAergic inputs onto abGCs has made it difficult to isolate sources of early synaptic GABA. Here, we examine a specific long-range GABAergic projection to the OB from the basal forebrain (Zaborszky et al., 1986; Nunez-Parra et al., 2013; Sanz Diez et al., 2019) and determine the developmental progression of basal forebrain GABAergic synaptic inputs onto abGCs. These experiments reveal an early developmental increase in GABAergic input from the basal forebrain onto abGCs, coinciding approximately with the abGC critical period. Furthermore, we find that basal forebrain GABAergic signaling is an important factor regulating the survival of adult-born neurons in the OB.

## Materials and Methods

### Animals

Mice were maintained on a 12 h light-dark cycle and were treated in compliance with the US Department of Health and Human Services and Baylor College of Medicine IACUC guidelines. Male and female littermate mice were used in all analyses and divided randomly between experimental conditions. All mice that underwent surgery were 2–4 months old. Mice used for electrophysiology and immunohistochemistry were 3–5 months old. Vgat-Cre (*Slc32a1*^tm2(cre)Lowl^, Stock: 028862) and Vgat^f/f^ (*Slc32a1*^tm1Lowl^, Stock: 012897) mice were originally purchased from Jackson Laboratories.

### Stereotaxic Injections and Viral Constructs

For all viral injections, mice were anesthetized with 4% isoflurane in O_2_ and maintained under anesthesia with 1–2% isoflurane in O_2_. Craniotomies were made over the sites of stereotaxic injections that were guided by Angle Two software (Leica) normalized to Bregma. To target the horizontal diagonal band of Broca (HDB), bilateral injections were made into the basal forebrain (from Bregma: ML ± 1.34 mm, AP +1.1 mm, DV −5.8 mm). The targeting of the HDB was verified in all cases by visualizing viral expression within the HDB. To target the RMS, bilateral injections were made at coordinates (from Bregma: ML ± 0.8 mm, AP +2.58 mm, DV −3.62 mm). To target the OB, craniotomies were made over the center of each bulb as identified by the eye. The injector tip was lowered to a depth of −0.8 mm from the dorsal surface of the OB. All viruses were packaged in-house and included: AAV-Ef1α-flex-mVenus Serotype retro2, AAV-Ef1α-flex-synaptophysin::eGFP-WPRE-hGHpA Serotype DJ8, AAV-Ef1α-flex-hChR2(H134R)-eYFP-WPRE-hGHpA, serotype 2/9, pLenti-CMV-tdTomato-WPRE, AAV-Ef1α-iCre-H2B::mVenus Serotype DJ8, AAV-Ef1α-H2B::mVenus, Serotype DJ8. HDB injections were done with 250 nl of the virus, RMS injections with 150 nl, and OB core injections with 500 nl.

### Immunohistochemistry

For immunohistochemistry, mice were deeply anesthetized then transcardially perfused with PBS followed by 4% PFA. Brains were removed and immersion fixed in 4% PFA overnight at 4°C. Brains were transferred to 30% sucrose and allowed to equilibrate, then they were frozen and sectioned at 40 μm on a cryostat (Leica). The sections were washed in 0.1% PBS-T, then incubated in a blocking solution composed of 10% normal goat serum, 0.3% PBS-T, and 3M glycine for 1 h at room temperature. For CHAT staining, the blocking buffer included 10% donkey serum replacing normal goat serum. Following blocking, slices were then incubated in primary antibody diluted in blocking buffer overnight at 4°C. The next day slices were washed 3× in 0.1% PBS-T then incubated in secondary antibody for 2 h at room temperature. Slices were then washed 3× in 0.1% PBS-T, transferred to 0.5× PBS, and mounted on glass slides with DAPI-containing mounting media (Southern Biotech). Primary antibodies used included: chicken ∝ GFP (1:1,000, Abcam, ab13970), Rabbit ∝ Ki67 (1:200, Vector, VP-RM04) and Goat ∝ CHAT (1:1,000, Chemicon, Ab144P). Secondaries used included Goat ∝ Chicken:488 (1:1,000, Invitrogen, A32931) and Goat ∝ Rabbit:546 (1:1,000, Invitrogen, A11035), and Donkey ∝ Goat:546 (Invitrogen A11056). Slices were imaged on a Leica SP8 Confocal with 10× or 20× dry objectives. GFP intensity across layers of OB slices was quantified in FIJI by taking intensity profiles of 10 pt wide line scans spanning the RMS to the surface of the OB. Five similarly-sized sections were quantified and averaged (Figure 2E). Cell counts were performed automatically in FIJI. Images were first automatically thresholded, then converted to binary images. Automated cell counting was carried out in each region using the “Analyze Particles” function in FIJI with uniform parameters across images. Regions of interest for cell counting were defined by hand using the DAPI channel compared to a reference atlas.

**Figure 1:**
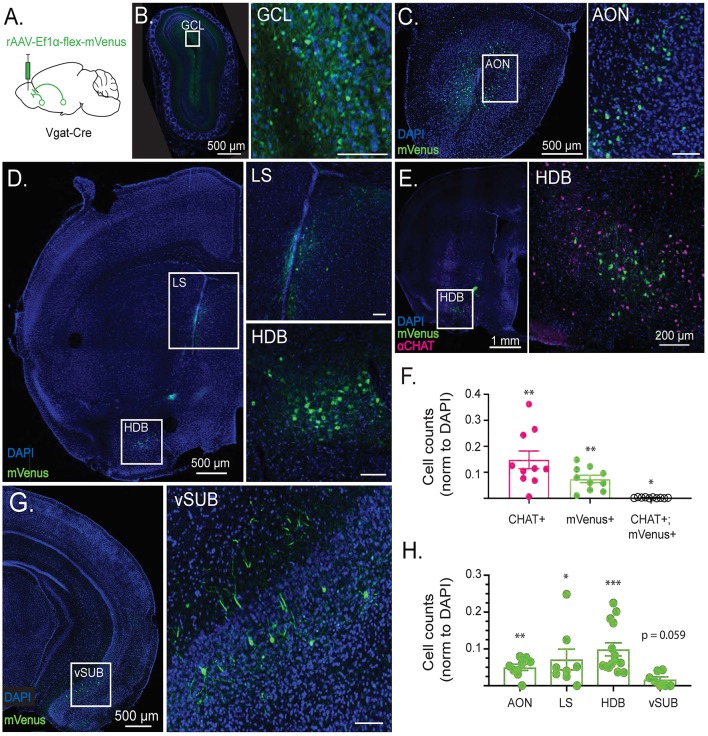
Cell type-specific retrograde tracing reveals long-range GABAergic projections to the olfactory bulb (OB). **(A)** Schematic depicting viral injection a retro2 serotype adeno associated virus expressing Cre-dependent mVenus (rAAV-Ef1α-flex-mVenus) into the OB of Vgat-Cre-expressing mice. Retrograde labeling reveals sources of synaptic input to the OB. **(B)** GABAergic retrograde labeling in OB with inset showing mVenus expression in the granule cell layer (GCL). **(C)** GABAergic retrograde labeling in a coronal section including the anterior olfactory nucleus (AON) with inset showing mVenus expression in lateral AON. **(D)** GABAergic retrograde labeling in a coronal section including the horizontal diagonal band of Broca (HDB) and lateral septum (LS) with insets showing mVenus expression in LS and HDB. **(E)** CHAT immunolabeling overlaid with GABAergic retrograde labeling in a coronal section including HDB, with inset showing lack of colocalization between CHAT+ and mVenus+ neurons in the HDB. **(F)** Quantification of CHAT+, mVenus+, and colocalized neurons in the HDB normalized to DAPI from single 40 μm thick sections. Points reflect cell counts from individual animals. *N* = 10 mice. Error bars are SE. One sample Wilcoxon rank-sum test. *p < 0.05, **p < 0.01. **(G)** Retrograde labeling in a coronal section including the ventral subiculum (vSUB) with inset showing sparse mVenus expression in vSUB. **(H)** mVenus+ cell counts by brain region from single 40 μm thick sections normalized to the number of DAPI+ cells in each region. Points reflect cell counts from individual animals. *N* = 8–14 mice. Error bars are SE. One sample Wilcoxon rank-sum test. *p < 0.05, **p < 0.01, ***p < 0.001. Scale bars are 100 μm unless otherwise specified.

**Figure 2:**
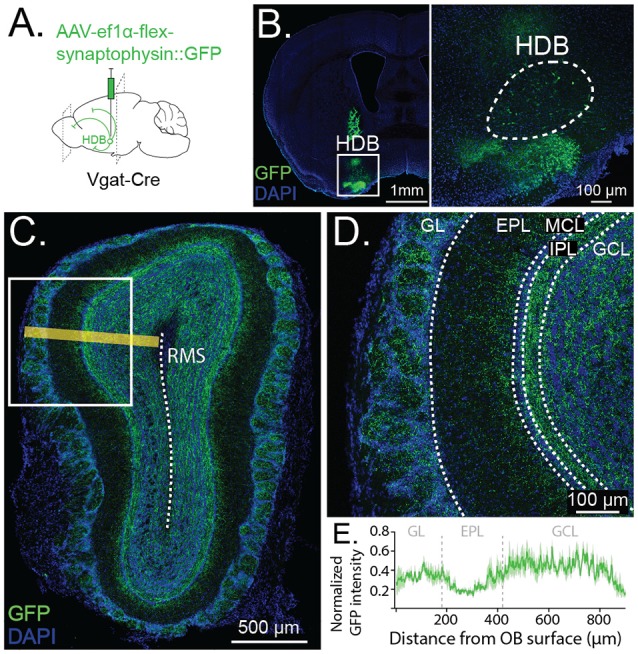
Anterograde tracing reveals GABAergic projections from HDB to GCL and glomerular layer (GL). **(A)** Schematic depicting viral injection of Cre-dependent synaptophysin fused to GFP (AAV-Ef1α-flex-synaptophysin::GFP) and anterogradely-labeled projections in Vgat-Cre-expressing mice. **(B)** Coronal section showing viral injection site and synaptophysin::GFP expression in HDB. Inset shows cell bodies expressing GFP in HDB (dashed lines) **(C)**. Anterograde labeling in OB with yellow line demonstrating orientation of the line scan plane from the OB surface to the rostral migratory stream (RMS, dashed line) for quantification of GFP intensity (shown in **E**). **(D)** Synaptophysin::GFP expression is dense throughout the GCL and GL, highest in the internal plexiform layer (IPL) and lowest in the external plexiform layer (EPL). **(E)** Quantification of GFP intensity along a line scan from the OB surface to 900 μm deep toward the RMS (shown in **C**). The dark green trace shows GFP intensity peak-normalized by the animal, averaged across five same-sized sections from five animals. The light green band shows SE. Dashed vertical lines show approximate borders of GL, EPL, and GCL.

### EdU Incorporation Assay

Two weeks before EdU injections, the HDB of 8–10 week old Vgat^f/f^ mice was targeted for viral injection of AAV-Ef1α-iCre-H2B::mVenus or AAV-Ef1α-H2B::mVenus. To measure the survival of birth-dated adult-born neurons, Vgat^f/f^ mice were then given a series of EdU injections (50 mg/ml stock in DMSO diluted to 5 mg/ml with sterile saline.) Mice were I. P. injected with an EdU dose of 5 mg/Kg 10 times over 9 h. Mice were then aged for 4 weeks in their home cages before harvesting (at 14–16 weeks), serial sectioning, and processing the brains for immunohistochemistry as described above.

For imaging EdU incorporation, a Click-iT Plus EdU Imaging Kit (Invitrogen) was used according to the packaged instructions. Briefly, brains were harvested, fixed, frozen and sectioned as described above. The sections were washed 2× in 0.1% PBS-T for 10 min, then for 20 min in 0.5% PBS-T at room temperature. Sections were washed 2× in PBS then incubated in the Click-iT reaction cocktail containing Alexa:647 picolyl azide for 30 min at room temperature protected from light. The sections were then washed 3× in PBS and mounted with DAPI-containing mounting media as described above. Following mounting, the slices were imaged on a Leica SP8 Confocal with a 10× air objective. To count EdU+ cells in the glomerular and granule cell layers (GCLs), regions were outlined manually in FIJI using the DAPI channel. Cell counts from each region were automated in FIJI as described above. EdU cells were counted from projections of 40 μm sections then normalized to the area of the region of interest from which they were counted to obtain the density (cells/mm^2^). Two to four sections were quantified from each animal and a nested *t*-test was used to compare the experimental and control groups to account for multiple sections being quantified in each animal.

### Dual mRNA Fluorescent *in situ* Hybridization

Dual mRNA *in situ* hybridization (ISH) was performed on 25 μm thick coronal sections cut from fresh-frozen Vgat^f/f^ mouse brains (aged 14–16 weeks) previously HDB-injected with virus expressing either Cre-mVenus or mVenus (control). We generated a digoxigenin (DIG)-labeled mRNA antisense probes against mVenus and fluorescein (FITC)-labeled mRNA against Vgat using reverse-transcribed mouse cDNA as a template and RNA DIG or FITC-labeling kits from Roche (Sigma). ISH was performed by the RNA *in situ* Hybridization Core at Baylor College of Medicine using an automated robotic platform as previously described (Yaylaoglu et al., 2005) with modifications of the protocol for double ISH. Modifications in brief (for buffer descriptions, see Yaylaoglu et al., 2005): both probes were hybridized to the tissue simultaneously. After the described washes and blocking steps the DIG-labeled probes were visualized using tyramide-Cy3 Plus (1/50 dilution, 15-min incubation, Perkin Elmer). After washes in TNT, the remaining HRP-activity was quenched by a 10 min incubation in 0.2 M HCl. The sections were then washed in TNT, blocked in TNB for 15 min before a 30 min room temperature incubation with HRP-labeled sheep anti-FITC antibody (1/500 in TNB, Roche). After washes in TNT the FITC-labeled probe was visualized using tyramide-FITC Plus (1/50 dilution, 15-min incubation, Perkin Elmer). Following washes in TNT the slides were stained with DAPI (Invitrogen), washed again, removed from the machine and mounted in ProLong Diamond (Invitrogen). *Vgat* expression in the HDB was quantified by counting *Vgat+* cells (identified by FISH) and normalizing to the total number of DAPI-stained cells in the HDB (Figures 5B,C). The HDB was outlined using the DAPI channel compared to a reference atlas.

**Figure 3:**
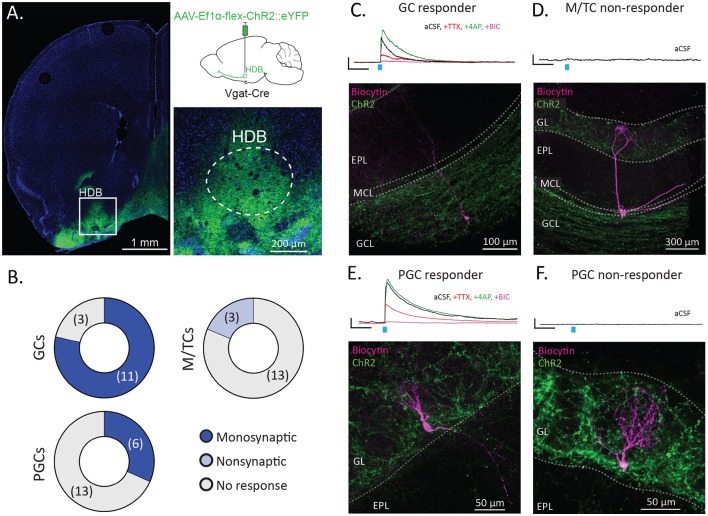
HDB GABAergic neurons preferentially synapse onto interneurons in the OB. **(A)** Coronal section shows the injection site with the inset showing dense membrane labeling of neurons in the HDB (dashed lines). The upper right panel shows schematically for injection of Cre-dependent Channelrhodopsin (AAV-Ef1α-flex-ChR2::eYFP) into the HDB of Vgat-Cre mice. **(B)** Pie charts showing proportions of GCs, M/TCs, and periglomerular cells (PGCs) responding to stimulation of ChR2-expressing HDB GABAergic terminals in OB slices. *N* = 11 mice total, 14–19 cells per group type. **(C)** Traces show an example of a light-evoked current from a granule cell (GC) responding to ChR2 stimulation (blue tick) in aCSF (black), and following serial bath application of tetrodotoxin (TTX, red), 4-aminopyridine (4AP, green), and bicuculline (BIC, purple). The image shows the corresponding biocytin cell fill spanning the GCL, mitral cell layer (MCL) and EPL. **(D)** The trace shows no response to light from a mitral/tufted cell (M/TC) in aCSF. The image shows the corresponding biocytin cell fill with the cell body and lateral dendrites in the MCL and apical dendrites in the GL. EPL and GCL labeled for orientation. **(E)** The trace shows a representative example of a light-evoked current from a PGC responding to ChR2 stimulation in aCSF and during serial additions of TTX, 4AP, and BIC. The image shows the corresponding cell fill with a cell body in and dendrites in the GL. **(F)** The trace shows no response to light from a non-responsive PGC in aCSF. The image shows the corresponding biocytin cell fill with the cell body and dendrites in the GL. All scale bars for traces are 5 pA and 100 ms.

**Figure 4:**
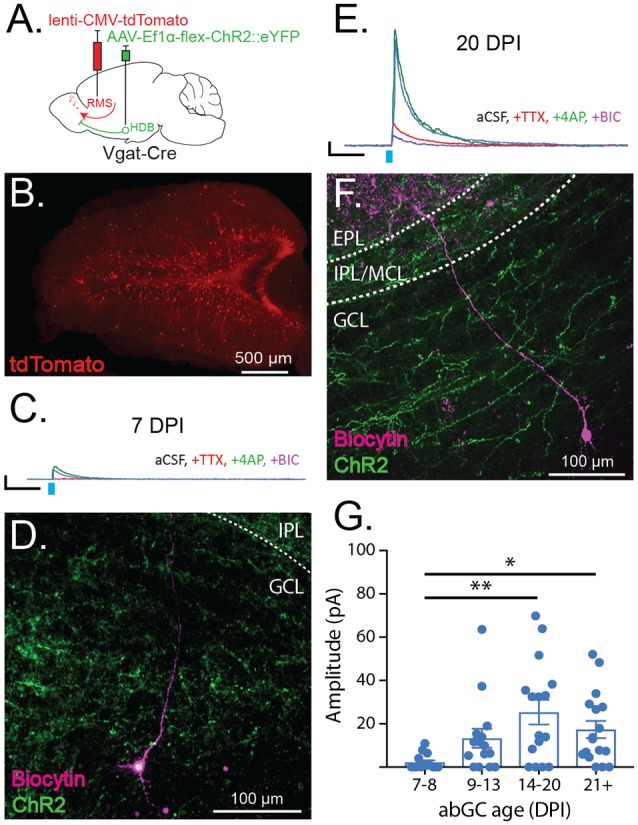
Adult-born granule cells (abGCs) receive early synaptic input from HDB GABAergic neurons. **(A)** Schematic showing viral injections for ChR2 expression (AAV-Ef1α-flex ChR2::eYFP) and adult-born neuron birth-date labeling (lenti-CMV-tdTomato). **(B)** Example of birth-dated adult-born neurons in a 150 μm thick horizontal section at 14 days post-RMS injection. **(C)** Traces show representative examples of currents evoked by ChR2 stimulation (light blue tick) in a labeled abGC at seven DPI in aCSF (blue), and following serial bath application of tetrodotoxin (TTX, red), 4-aminopyridine (4AP, green), and bicuculline (BIC, purple). Scale bar X and Y axes are 100 ms and 10 pA. **(D)** Cell fill corresponding to the trace in **(C)** showing cell body in deep GCL and dendrite extending toward the IPL. **(E)** Same as **(C)** for abGC at 20 DPI. **(F)** Cell fill corresponding to the trace in **(E)** showing cell body in GCL and dendrite spanning IPL and EPL. **(G)** Amplitudes of light-evoked currents from individual birth-dated neurons at different days post RMS injection (DPI) binned by age. *N* = 23 mice, 60 cells. Error bars are SE. Points reflect current amplitudes (pA) from individual cells in aCSF. **p < 0.01, *p < 0.05, one-way ANOVA with Tukey’s *post hoc* test for multiple comparisons.

**Figure 5:**
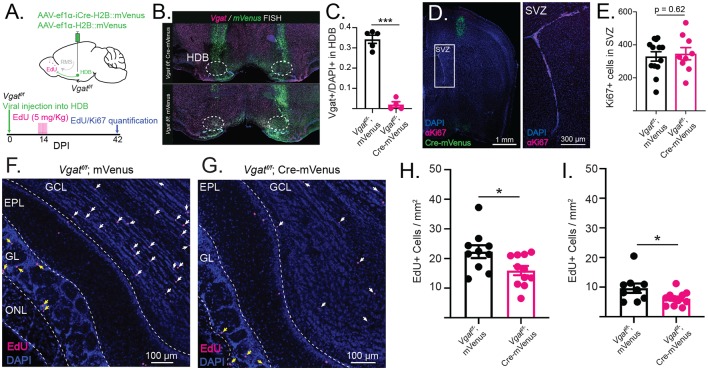
Conditional knockout of VGAT in the HDB reduces the survival of adult-born neurons in GCL and GL. **(A)** Schematic showing injection of Cre-mVenus or mVenus control virus into the HDB of Vgat^f/f^ mice. The timeline shows the experimental design with viral injection into HDB of Vgat^f/f^ mice followed 2 weeks later by I. P. injections of EdU and 6 weeks later by quantification of EdU incorporation into OB and Ki67 expression in the subventricular zone (SVZ). **(B)** Dual-color fluorescent *in situ* hybridization (FISH) confirms that VGAT is knocked out in Cre-expressing cells after viral injection of Cre into the HDB of Vgat^f/f^ mice (top) whereas *Vgat* is expressed in cells infected with the control mVenus virus injected into the HDB of Vgat^f/f^ mice (bottom). **(C)** Quantification of *Vgat*+ cell density in the HDB of cre-injected (magenta) and control-injected (black) Vgat^f/f^ mice. *N* = 9 mice, 18 sections. Nested *t*-test, ***p < 0.001. **(D)** Coronal section from a Vgat^f/f^ mouse HDB injected with Cre-mVenus (green) and stained for Ki67 (magenta) to label proliferating progenitor cells in the SVZ. The inset shows Ki67+ cells in the SVZ. **(E)** Quantification of Ki67+ cells in the SVZ of Vgat^f/f^ mice HDB-injected with Cre-mVenus or mVenus control viruses (black). Points represent values from individual mice. *N* = 23 mice, 37 sections. Nested *t*-test. **(F)** OB sections from Vgat^f/f^ mice injected into the HDB with Cre-mVenus showing EdU incorporation (magenta). White arrows mark EdU+ cells in the GCL. Yellow arrows mark EdU+ cells in the GL. ONL: olfactory nerve layer. **(G)** Same as **(F)**, but in Vgat^f/f^ mice injected with mVenus virus (control). **(H)** Quantification of EdU+ cell density in GCL of 40 μm OB sections from Vgat^f/f^ mice HDB-injected with Cre (magenta) or mVenus (black; **I**). Same as **(H)** but quantified in the GL. Points represent values from individual mice. *N* = 21 total mice, 69 sections. Error bars are SE. Nested *t*-test, *p < 0.05.

### Electrophysiology and Optogenetic Circuit Mapping

For slice electrophysiological recording experiments, mice were deeply anesthetized with isoflurane then transcardially perfused with ice-cold artificial cerebrospinal fluid (aCSF) solution containing (in mM): 125 NaCl, 2.5 KCl, 1.25 NaH_2_PO_4_, 1 MgCl_2_, 2 CaCl_2_, 25 glucose, and 25 bicarbonate (pH 7.3, 295 mOsM). Brains were removed and transferred into ice-cold cutting solution containing (in mM): 2.5 KCl, 1.25 NaH_2_PO_4_, 10 MgSO_4_, 0.5 CaCl_2_, 234 sucrose, 11 glucose, and 26 bicarbonate. Cutting solution was continuously bubbled with 95% CO_2_/5% O_2_. For OB coronal sections, brains were blocked coronally through the somatosensory cortex and embedded in 1.5% low melting point agarose. Agar-embedded brains were immediately submerged in oxygenated cutting solution on a Leica VT1200 vibratome. Three-hundred micrometers coronal sections were made at a cutting speed of 0.4 mm/s. Slices were removed to a slice recovery chamber of oxygenated aCSF at 37°C for at least 30 min. Following recovery, slices were slowly returned to room temperature for 30 min before recording.

For whole-cell voltage-clamp recordings, slices were submerged in a recording chamber and continuously perfused with room temperature oxygenated aCSF at ~2 ml/min. For recording a mixed population of GCs, Mitral and Tufted cells, and Juxtaglomerular cells, cells were visualized with DIC optics (Olympus BX50WI). Cells were identified by their location within the OB, their unique morphologies, and intrinsic properties. For recording birth-dated adult-born GCs, cells were identified after viral labeling in the RMS using fluorescence imaging. They were then visualized for whole-cell recording using DIC optics. Once visualized, cells were whole-cell patched in voltage-clamp configuration. Recording electrodes (3–7 mOsM) were pulled from thin-walled borosilicate glass capillaries (inner diameter: 1.1 mm, outer diameter: 1.5 mm) with a horizontal micropipette puller (Sutter Instruments). Voltage-clamp internal solution contained (in mM): 120 Cs Methanesulfonate, 6 CsCl, 20 HEPES, 1 EGTA, 0.2 MgCl_2_, 10 phosphocreatine, 4 MgATP, 0.4 NaGTP (with 0.4% biocytin by weight, pH to 7.3 with CsOH, 285 mOsM). Recordings were made using PClamp software (Axon) with an Axon MultiClamp 700B amplifier digitized at 10 kHz (Axon Digidata 1440A).

For optogenetic circuit mapping of basal forebrain GABAergic inputs, patched cells were first voltage-clamped at −65 mV to record baseline membrane properties. To check for the presence of a light-evoked inward current, channelrhodopsin was activated by full-field illumination from a filtered xenon light source filtered to (Olympus, U-N41020). The onset and duration of light stimulation was controlled through ClampEx software by a mechanical shutter (Sutter). No inward currents were ever observed when stimulating release from HDB GABAergic terminals onto abGCs, GCs, PGCs, or M/TCs. Patched cells were then voltage-clamped at 0 mV (adjusted for junction potential) to reveal outward currents. If a light-evoked outward current was observed in aCSF, then TTX (1 μM), 4AP (0.5 μM), and bicuculline (BIC, 10 μM) were serially bath-applied to verify: (1) the action potential-dependence; (2) the monosynaptic nature; and (3) the GABA receptor-dependence of the evoked current. All cells were dialyzed with 0.15–0.4% biocytin for the duration of the recording and patched neurons were saved for *post hoc* staining, imaging, and reconstruction. After recordings, electrodes were withdrawn slowly allowing the cells to reseal and form an outside-out patch. Slices were then allowed to equilibrate in the recording chamber for 5 min before being transferred to 4% PFA.

After patching and cell-filling, slices were fixed overnight in 4% PFA at 4°C. Slices were then washed 3× in 0.1% PBS-T for 30 min each. After washing, the slices were incubated in 10% normal goat serum blocking buffer for 2 h at room temperature. Slices were then incubated in streptavidin conjugated to Alexa:647 (1:500, Invitrogen) overnight at 4°C. The next day, slices were washed 3× in 0.1% PBS-T for 30 min, then mounted on glass slides using 500 μm spacers (Electron Microscopy Sciences) filled with mounting media without DAPI (Southern Biotech). Slices were imaged on a Leica SP8 confocal with 10× and 20× objectives. Z stacks of filled cells were reconstructed in FIJI.

During all recordings, access resistance was continuously monitored, and cells, where the access resistance changed by more than 20%, were excluded from the analysis. All traces were baseline subtracted and filtered with a Gaussian filter in ClampFit (PClamp). Traces were then exported to MATLAB where cell-intrinsic and evoked-current properties were quantified with custom scripts.

### Experimental Design and Statistical Analysis

Data were analyzed in Prism8 (Graphpad). For comparisons of means between two groups, two-tailed student’s *t*-tests were performed. For comparisons of means to a hypothetical mean, one sample Wilcoxon rank-sum tests were used against a hypothetical mean of 0. For comparisons between multiple groups, one-way ANOVAs were used followed by Tukey’s *post hoc* correction for multiple comparisons. *P*-values less than 0.05 were considered to be significant. For currents evoked in adult-born neurons, current amplitudes were grouped by the age of the recorded neuron. Means from age groups were compared with a one-way ANOVA followed by Tukey’s *post hoc* correction for multiple comparisons. EdU+, mVenus+, Ki67+, CHAT+, and DAPI+ cell counts were automated using a macro in FIJI. Two to four slices were imaged and quantified from each animal. Cell counts were compared between experimental and control conditions using a nested *t*-test where sections from each animal are averaged and N is the number of animals.

## Results

### The Olfactory Bulb Receives Long-Range GABAergic Input From the Horizontal Limb of the Diagonal Band of Broca

Given the influence that long-range GABAergic signaling has over both acute circuit function and neuronal development, we first sought to identify GABAergic neuron populations that project to the OB. To selectively localize GABAergic neurons projecting to the OB, we used conditional viral genetic techniques to retrogradely label GABAergic neuronal populations that send projections to the OB. First, we injected OBs of Vgat-Cre-expressing mice with a retrograde Cre-dependent mVenus (rAAV-Ef1α-flex-mVenus), to preferentially infect presynaptic terminals (Figure 1A) and selectively label GABAergic neurons that project to the OB.

Three weeks after viral injection, labeled cells were imaged in serial coronal sections. The retro label mVenus was highly expressed within the OB, reflecting local GABAergic populations (Figure 1B). Additionally, retro labeling revealed GABAergic projections to the OB from the medial and lateral anterior olfactory nucleus (AON; Figure 1C), the horizontal limb of the diagonal band of Broca (HDB), and the lateral septum (LS; Figure 1D). Notably, a subset of GABAergic projection neurons in the HDB co-transmit acetylcholine (Granger et al., 2016). To determine the extent to which the observed population of OB-projecting HDB GABAergic neurons were cholinergic co-transmitting neurons, we stained OB retro-labeled sections for the cholinergic marker CHAT (Figure 1E). Quantification of the mVenus retro label and αCHAT immunofluorescence colocalization revealed that the vast majority of retro-labeled GABAergic neurons in the HDB (95.9% ± 1.5%) do not co-transmit acetylcholine (Figure 1F). The small but significant population of retro-labeled HDB neurons that do co-express CHAT are likely the co-transmitting neurons projecting specifically to internal plexiform layer (IPL) deep short axon cells (Case et al., 2017). In addition to the AON, LS, and HDB populations, retro-labeling also revealed a previously-unidentified sparse population of GABAergic neurons in the ventral subiculum (vSUB) that project to the OB (Figure 1G). However, while OB-projecting GABAergic neurons comprised significant populations of AON, LS, and HDB neurons, OB-projecting GABAergic neurons in vSUB were merely trending toward significance as a population, reflecting their relative sparseness (Figure 1H). Importantly the AON, HDB, LS, and vSUB all receive reciprocal glutamatergic projections from the OB and other olfactory areas (Shipley and Adamek, 1984). Together, these areas form an established network of brain regions involved in olfactory processing which, through GABAergic projections to the OB, may exert direct, top-down control over the earliest stages of olfactory processing.

Having established that a relatively dense population of HDB neurons send GABAergic projections to the OB, we next sought to determine the anatomical and functional specificity of these projections within the OB. To determine where within the OB the HDB GABAergic neurons project to, we anterogradely labeled HDB GABAergic neurons and imaged terminals in the OB. To anterogradely label HDB GABAergic neurons, we injected an AAV expressing Cre-dependent synaptophysin fused to eGFP (AAV-Ef1α-flex-synaptophysin::eGFP) into the HDB of Vgat-Cre mice (Figures 2A,B). The fusion of eGFP to membrane-bound synaptophysin allowed visualization of fine processes and axon terminals. While, under these circumstances, a larger area of the basal forebrain is infected with the virus than just the HDB (Figure 2A), our earlier retrograde tracing demonstrates that the only the clustered population of GABAergic neurons in the HDB project to the OB (Figure 1D). Specifically, we did not identify GABAergic projections from the olfactory tubercle to the OB, in agreement with a recent study (In’t Zandt et al., 2019), but in contrast to earlier work examining non-cell type-specific projections (Wesson and Wilson, 2011). While our current data strongly suggest that GABAergic synaptic terminal labeling in the OB results from HDB expression of synaptophysin::eGFP, we cannot exclude that some terminal labeling in the OB results from viral expression in the surrounding areas. Confocal imaging and tiled reconstructions of OB slices showed that HDB GABAergic neurons project primarily to the GCL (GCL), the IPL, and the glomerular layer (GL; Figure 2C). Within the GCL, projections were most dense in the superficial layers near the IPL (Figures 2D,E). Together these data suggest that HDB GABAergic neurons selectively project to subsets of OB neurons in the GCL and GL.

### GABAergic Projections From the Horizontal Limb of the Diagonal Band of Broca Synapse Onto Interneurons in the Granule Cell Layer and the Glomerular Layer of the Olfactory Bulb

Having observed that HDB GABAergic neurons selectively project to the GCL and GL within the OB, we next sought to determine the functional connectivity of HDB GABAergic neurons onto neuronal subtypes within the OB. Towards this, we combined slice electrophysiology with optogenetic stimulation to assay monosynaptic connections from basal forebrain GABAergic neurons onto a variety of OB neuronal subtypes (Petreanu et al., 2009). We injected Vgat-Cre mice with AAV-Ef1α-flex-ChR2::eYFP in the HDB. Two weeks later we made acute brain slices from the OB for electrophysiology and made whole-cell patch recordings from mitral and tufted cells (M/TCs), granule cells (GCs), and periglomerular cells (PGCs). Cells were voltage-clamped at 0 mV to isolate light-evoked GABAergic currents. In the case that a light-evoked current was observed in the patched cell, TTX, 4AP, and Bicuculline were serially applied to the bath to assess whether the current was monosynaptic and GABAergic. During recording, cells were filled with biocytin for *post hoc* reconstruction to confirm their cellular identities.

Optogenetic circuit mapping revealed that GCs receive robust input from HDB GABAergic neurons in agreement with a previous report (Nunez-Parra et al., 2013). Eleven of fourteen GCs showed monosynaptic input from HDB GABAergic neurons (Figures 3A–C). Additionally, we found that M/TCs receive no monosynaptic input and limited non-monosynaptic input from HDB GABAergic neurons. Thirteen of 16 M/TCs showed no light-evoked current (Figures 3B,D). The remaining three M/TCs showed a weak, light-evoked current that was attenuated by TTX but not potentiated by 4AP, indicating that the current was not directly monosynaptic. The remaining current in TTX and 4AP was blocked by Bicuculline (BIC) indicating that it relied on GABA receptors. PGC (PGC) connectivity was heterogeneous, with 6 of 19 PGCs receiving monosynaptic input from HDB GABAergic neurons, while 13 of 19 received no input (Figures 3B,E,F). This is in agreement with a recent report demonstrating monosynaptic connections onto Group 1 PGCs, a category that includes superficial short axon cells (Sanz Diez et al., 2019). Together, these data suggest that HDB GABAergic inputs preferentially target OB interneurons in the GCL and periglomerular regions of the GL.

### Immature Adult-Born Granule Cells Receive Early Synaptic GABAergic Input From the Horizontal Limb of the Diagonal Band of Broca

The observed preferential targeting of OB interneurons by HDB GABAergic projections led us to question whether HDB GABAergic neurons synapse onto immature adult-born interneurons and whether this input may influence their circuit integration and survival. To determine whether, and precisely when immature abGCs receive GABAergic input from the HDB, we performed optogenetic circuit mapping on abGCs of different ages. First, we injected the HDB of Vgat-Cre mice with flex-ChR2 to allow the optogenetic stimulation of GABAergic terminals. Two weeks later, we labeled adult-born neurons by injecting a lentivirus expressing tdTomato into the RMS (Figure 4A). Under these circumstances, only abGCs in the RMS on the day of the injection become infected by the lentivirus and express tdTomato in the OB after the migration (Figure 4B).

By making acute slices from the OB at different time points after RMS injections, we recorded evoked GABAergic currents onto precisely birth-dated abGCs. We then quantified the extent of connectivity as the amplitude of the light-evoked, monosynaptic current onto birth-dated abGCs. These experiments revealed that monosynaptic GABAergic input onto abGCs first emerges at 7 days post-injection (DPI; Figures 4C,D; 7–8 DPI = 2.19 ± 6.09 pA) and increases between seven and 20 DPI (Figures 4E,F; 14–20 DPI = 25.37 ± 5.99 pA). After 20 DPI, HDB GABAergic input on to abGCs became stable, matching evoked currents onto resident and mature GCs (Figure 4G; 21 + DPI = 17.36 ± 5.90 pA). These data indicate that abGCs receive monosynaptic GABAergic input from the HDB early in their development and that this input gradually increases through the 2nd and 3rd week of abGC maturation, during their critical period.

### GABAergic Projections From the Horizontal Limb of the Diagonal Band of Broca Promote the Survival of Adult-Born Neurons in the Olfactory Bulb

The observation that immature abGCs receive early GABAergic input from the HDB led us to examine whether these inputs influence abGC circuit integration into the OB. To address this, we eliminated GABA release from neurons in the HDB by injecting an AAV expressing Cre and mVenus into the HDB of conditional floxed Vgat mice (*Slc32a1*^tm1Lowl^, Jax Stock: 012897; Vgat^f/f^; Cre-mVenus, Figure 5A). Vgat^f/f^ littermates were injected with an AAV expressing mVenus as controls (Vgat^f/f^; Ef1α-mVenus). The efficient KO of VGAT in the HDB was confirmed with dual-color FISH labeling mVenus and Vgat mRNA (Figure 5B). Vgat^f/f^ animals injected with the control mVenus-expressing virus showed significant colocalization of mVenus and Vgat mRNA. Vgatf^/f^ mice injected with the experimental Cre-mVenus virus, however, showed no colocalization of *mVenus* and *Vgat* mRNA. Within the HDB, *Vgat* expression was effectively knocked out (Figure 5C), indicating high-efficiency viral infection and consistent targeting across animals (*N* = 9 mice, 18 sections, nested *t*-test, *p* < 0.001). After 2 weeks, mice were treated with EdU to label dividing cells. Four weeks later (6 weeks after HDB viral injections) we measured Ki67 protein expression in the SVZ as a marker for abGC progenitor proliferation (Figure 5D), and EdU incorporation in the OB as a marker of adult-born neuron survival (Figures 5F,G). Additionally, HDB targeting was confirmed by visualizing mVenus expression in the HDB. Quantification of Ki67+ cells in the SVZ revealed no difference in adult-born neuron progenitor proliferation (Figure 5E). However, quantification of EdU incorporation into the OB revealed a significant decrease in adult-born neuron survival after knockout of VGAT in the HDB. To distinguish between the effects of HDB GABAergic input on adult-born neurons in the GCL (mainly abGCs) and other adult-born neurons in the GL (GABAergic PGCs), we quantified the density of EdU+ neurons in the GCL and GL separately. Quantification revealed fewer EdU+ cells in the GCL when VGAT was removed from HDB neurons (*N* = 11 mice, 37 sections sections) compared to controls (*N* = 10 mice, 32 sections, *p* = 0.01, nested *t*-test, Figure 5H). The decrease in adult-born neuron survival was also observed in the GL (nested *t*-test, *p* < 0.05, Figure 5I), suggesting that HDB GABAergic input promotes the survival of adult-born GCs and PGCs. Together, these findings suggest a mechanism by which activation of HDB GABAergic signaling controls circuit-level plasticity in the OB and drives lasting changes in the networks that govern the initial stages of olfactory processing.

## Discussion

GABA is a key developmental signal in adult neurogenesis, where its role is analogous in many ways to the role of GABAergic signaling in embryonic and early postnatal development (Stewart et al., 2002; Wang et al., 2003; Bolteus and Bordey, 2004; Liu et al., 2005; Pallotto et al., 2012). As in embryonic and early postnatal development, GABA is depolarizing in developing adult-born neurons, with the switch in GABA polarity occurring within 24 days of neurogenesis (Belluzzi et al., 2003; Carleton et al., 2003). This time-frame corresponds with the critical period of adult-born neuron development, during which they form synaptic connections and are fated for survival or death (Yamaguchi and Mori, 2005). As adult-born neurons integrate into existing circuits in the OB, they first receive GABAergic synapses (Panzanelli et al., 2009; Pallotto et al., 2012). This pattern matches the developmental progression of synaptogenesis in embryonic and postnatal developing neurons, where it is suggested that the depolarizing nature of early GABAergic synapses promotes subsequent morphological and functional maturation (Ganguly et al., 2001; Cancedda et al., 2007). Given the developmental importance of early GABAergic signaling, we sought to identify early synaptic input from long-range GABAergic projections and determine their influence on developing adult-born neurons in the olfactory system.

To date, early sources of synaptic GABA, the polarity of the GABA currents, and the timing of specific GABAergic inputs onto developing adult-born neurons in the OB remain poorly-understood. Historically this has been difficult to study given the diversity of local and long-range GABAergic signals within the OB, and the limited time window of the adult-born neuron critical period. Here we demonstrate that the OB receives robust, direct GABAergic projections from the HDB, which preferentially synapse onto subpopulations of OB interneurons that include abGCs. Notably, we found that abGCs receive input from HDB GABAergic neurons early in their development. These findings suggest that earlier retrograde tracing with the rabies virus may have systematically missed inputs to very immature abGCs which likely act at unconventional, immature, or sparse synapses (Deshpande et al., 2013). In the current study, by determining a developmental timeline of functional connectivity, we have revealed that long-range HDB GABAergic signaling onto abGCs emerges early during their critical period and that basal forebrain GABAergic signaling is important for the survival of adult-born neurons through the critical period in the OB.

Mapping projections to the OB has previously revealed robust centrifugal projections from the piriform cortex, basal forebrain, and hippocampus. Importantly, our cell-type-specific retrograde tracing reveals that a substantial population of these projections are from GABAergic neurons residing in the HDB, AON, and LS. Additionally, we identify a sparse population of GABAergic neurons projecting from vSUB to the OB, specifically implicating vSUB (in addition to HDB, LS, and AON) in the top-down regulation of olfactory processing. It is also notable that the pattern of GABAergic labeling in the AON described here matches the non-cell type-specific pattern of retrograde labeling observed by Shipley and Adamek (1984) in the contralateral AON. This raises the possibility that GABAergic projections to the OB from the AON arise uniquely from the contralateral AON and may, therefore contribute to bilateral olfactory comparisons and odor localization *via* top-down regulation of OB circuits (Kikuta et al., 2010; Jones and Urban, 2018). However, future work will be necessary to determine the extent to which contralateral projections from AON to OB are GABAergic and how they influence OB circuit activity.

In the context of sensory processing and experience-dependent plasticity, the dense projections from the HDB to the OB are particularly notable. Different populations of neurons within the HDB have been implicated in a variety of behavioral states like wakefulness, attention, appetite, and aversion, as well as in complex processing like the response to reinforcement learning (Anaclet et al., 2015; Hangya et al., 2015; Kim et al., 2015; Lin et al., 2015; Herman et al., 2016; Patel et al., 2019). At the same time, HDB neurons respond to multimodal sensory stimuli and send reciprocal projections to sensory processing centers where they influence network activity (Rye et al., 1984; Gaykema et al., 1990; Goard and Dan, 2009; Devore et al., 2015; Chaves-Coira et al., 2016; Do et al., 2016; Kim et al., 2016; Patel et al., 2019; Sanz Diez et al., 2019). From this, it follows that cumulative output from HDB would reflect the overall extent of salient sensory experience. HDB circuits are also in a position to acutely integrate incoming sensory signals with internal state information. Accordingly, the HDB is a source of top-down regulation for sensory systems including olfaction (Sarter and Bruno, 1997; Lau and Salzman, 2008; Rothermel et al., 2014). Along these lines, our current data showing that HDB GABAergic neurons synapse onto mature and immature GABAergic neurons in the OB support a common pattern whereby GABAergic projection neurons preferentially contact GABAergic neurons in target brain regions (Freund and Meskenaite, 1992; Gracia-Llanes et al., 2010; McDonald et al., 2011; Sanz Diez et al., 2019). This motif is important given that inhibitory inputs onto inhibitory interneurons are potent points of control over the population activity, capable of driving oscillations associated with brain states like wakefulness and conscious cognition (Kim et al., 2015). Thus, HDB GABAergic projections are particularly well-suited to control olfaction, sensory processing, and even cognition, in a top-down state-dependent manner.

In contrast to the acute regulatory potential of HDB GABAergic projections, their impact on adult-neurogenesis is capable of affecting lasting structural changes in the OB. Adult-neurogenesis confers a unique form of plasticity to the olfactory system which allows OB circuits to adapt and respond to different sensory environments throughout life. Adult-born neuron survival and integration is strongly influenced by environmental factors like sensory enrichment, deprivation, and olfactory learning. It is potently enhanced by complex sensory experiences like olfactory enrichment and learning. Given the role of the HDB in processing multimodal sensory and state information, early GABAergic input from the HDB may serve as an indicator of complex sensory experience above and beyond local OB circuit activity. At the same time, it is important to note that in the current experiment, *Vgat* was not only removed from HDB neurons projecting to the OB. But also from a localized, yet indiscriminate swath of neurons within the basal forebrain. Therefore, it is possible that knocking out GABAergic transmission from other projection populations, and reducing local GABAergic signaling within the basal forebrain may indirectly influence abGC survival. Nevertheless, our data support the conclusion that GABAergic signaling from the basal forebrain promotes the survival of adult-born neurons in the OB through the critical period. Also, the early influence of HDB GABAergic projections on developing adult-born neurons provides a novel mechanism linking state and experience-dependent signals in the basal forebrain to lasting structural plasticity in OB circuits.

## Data Availability Statement

The datasets generated for this study are available on request to the corresponding author.

## Ethics Statement

The animal study was reviewed and approved by Baylor College of Medicine IACUC.

## Author Contributions

EH designed the study, carried out experiments, analyzed the data, and wrote the manuscript. JS carried out experiments and analyzed data. BA provided input on experimental design, data analysis, and manuscript editing.

## Conflict of Interest

The authors declare that the research was conducted in the absence of any commercial or financial relationships that could be construed as a potential conflict of interest.
